# Notch Effect in Acrylonitrile Styrene Acrylate (ASA) Single-Edge-Notch Bending Specimens Manufactured by Fused Filament Fabrication

**DOI:** 10.3390/ma17215207

**Published:** 2024-10-25

**Authors:** Sergio Cicero, Fabrizia Devito, Marcos Sánchez, Sergio Arrieta, Borja Arroyo

**Affiliations:** 1 Laboratory of Materials Science and Engineering (LADICIM), Departamento de Ciencia e Ingeniería del Terreno y de los Materiales, University of Cantabria, Avenida de los Castros, 44, 39005 Santander, Spain; 2 Department of Mechanical, Mathematics and Management (DMMM), Polytechnical University of Bari, 70126 Bari, Italy; fabrizia.devito@poliba.it; 3 Department of Sciences, Technologies and Society, University School for Advanced Studies Pavia IUSS, 27100 Pavia, Italy; 4 TECNALIA, Basque Research and Technology Alliance (BRTA), Mikeletegi Pasealekua 2, 20009 Donostia-San Sebastián, Spain; 5 Departamento de Ingeniería Geografica y Tecnicas de Expresion Grafica, Escuela Técnica Superior de Ingenieros de Caminos, Canales y Puertos, Universidad de Cantabria, Avenida de los Castros, 44, 39005 Santander, Spain

**Keywords:** fused filament fabrication, acrylonitrile-styrene-acrylate, notch effect, fracture

## Abstract

This paper analyses the notch effect in the fracture behaviour of acrylonitrile–styrene–acrylate (ASA) material manufactured by fused filament fabrication (FFF). The research is performed on 72 single-edge-notch bending (SENB) specimens containing U-notches with nominal notch radii varying from 0 mm (crack-like defects) up to 2.0 mm, and fabricated with three different raster orientations (0/90, 45/−45, 30/−60). Apparent fracture toughness values are obtained for the different conditions and the resulting notch effect is analysed through the Theory of Critical Distances. A fractographic analysis is also performed using Scanning Electron Microscopy (SEM) in order to justify the fracture (macroscopic) behaviour from the observed fracture micromechanisms. The notch effect observed in the three ASA raster orientations is very similar, and lower than that observed in other FFF polymeric alternatives (ABS, PLA).

## 1. Introduction

Nowadays, Additive Manufacturing (AM) is a widely used technology enabling the generation of complex geometries through a simple process and has thus become an essential tool in Industry 4.0 [1,2]. AM includes various technologies, classified into seven categories by ASTM International [3], depending on the different material, physical state and energy conditions used during the printing process [4]: binder jetting, directed energy deposition, material extrusion, material jetting, power bed fusion, sheet lamination and vat photopolymerization. One of the most well-known AM technologies is Fused Deposition Modelling (FDM), first proposed and trademarked by Stratasys [5]. This technology is also known by the generic term Fused Filament Fabrication (FFF), and it is part of the material extrusion (MEX) technology [3]. FFF technology consists of extruding a heated plastic filament through a nozzle tip. The extruded material is deposited layer by layer to build the final component following a predefined digital model. FFF technology requires materials with adequate printability, while maintaining their functionality, which in practice involves a low coefficient of thermal expansion (CTE), an adequate fluid index and sufficiently high mechanical properties [6,7]. Among its main limitations are the rough surface finish, the (possible) low resulting strength (especially in the Z-direction) and the anisotropy of the printed parts, together with the risk of warping during the printing process [8,9].

Different materials can be printed using FFF, including polymers, metals and composites. When it comes to polymers and polymer matrix composites, acrylonitrile–butadiene–styrene (ABS) and polylactic acid (PLA) are the most widely used materials. The authors have previously analysed the mechanical performance of these two materials, including the notch effect in fracture conditions [10,11]. Given the relatively lower mechanical performance of polymers, and particularly of FFF polymers, when compared to traditional structural materials (e.g., metallic alloys), a typical strategy to enhance their behaviour consists of combining a polymeric matrix with one or more reinforcements to produce new or optimised composite materials with improved properties. Several polymer-matrix composites are usually obtained by the addition of fibres (e.g., glass fibres (GF) or carbon fibres (CF)), which play a reinforcing role and reduce the CTE [12]. On other occasions, the polymeric matrix is reinforced by adding nano-reinforcements (e.g., graphene, graphene oxide, carbon nanotubes, nano-clays, etc.) [13,14,15,16], particles (e.g., Al203) or a combination of fibres, particles and/or nano-reinforcements.

Through FFF technology, part quality, production efficiency and mechanical properties are affected by a large number of printing parameters such as nozzle temperature, layer thickness, bed temperature, printing speed, infill rate, build and layer orientation, type of infill structure, size of the air gap, etc. [17,18,19]. In this context, the literature presents numerous works analysing how tensile properties change with printing parameters (e.g., [17,18,19,20]). Generally, when dealing with (strictly) polymeric FFF materials, tensile properties are higher when the raster orientation coincides with the loading direction, when the infill level is 100%, the printing speed is moderate and the layer height is reduced. On the other hand, the works analysing the effects of printing parameters on the final fracture behaviour are more limited.

In this context, the acrylonitrile–styrene–acrylate (ASA) terpolymer emerges as a promising engineering alternative, printed directly or as the matrix in different composite materials. ASA shares a core–shell structure resembling that of ABS, with the difference that acrylate rubber replaces butadiene rubber. This substitution helps mitigate butadiene rubber’s physical or chemical ageing by preventing the degradation of the C=C double bond in the ABS backbone [21,22]. Thus, in outdoor or harsh environments, ABS can degrade over time, changing its mechanical properties, appearance and dimensional stability. This degradation can occur due to UV exposure, high temperatures, humidity and chemicals. ASA, however, exhibits a remarkable resistance to weathering agents and, particularly, has better UV resistance, making it more suitable for external applications where ABS could degrade more quickly. It also presents heat and oil resistance, preventing discoloration. Additionally, thanks to its unique core–shell structure, ASA possesses numerous other good qualities, including good mechanical properties (including toughness) and both dimensional and thermal stability, making it suitable for various outdoor applications (e.g., automotive, gardening) [23,24,25]. ASA also exhibits significantly greater resistance to environmental stress cracking when compared to ABS, especially against alcohols and many detergents. Consequently, ASA is generally preferred in the aerospace, automotive, and marine sectors [26].

Concerning the mechanical performance of additively manufactured ASA, there are a number of papers analysing and optimising the tensile behaviour of this material (e.g., [26,27,28,29,30]). In general, they reveal that the tensile properties (yield strength, tensile strength, elastic modulus) are comparable to those developed by ABS and PLA, with tensile strength values typically ranging between 40 and 50 MPa when printing parameters are optimised. Other works, where optimising printing parameters is not necessarily the objective of the research, present lower strengths, ranging between 16 and 30 MPa (e.g., [31,32,33]). When dealing with fracture behaviour, the works investigating it in additively manufactured ASA are scarce (e.g., [34,35,36,37]), and they generally analyse impact toughness, with very little reported information (e.g., [37]) regarding the specific fracture toughness value of this particular material. Finally, regarding the notch effect, to the knowledge of the authors, there is no reported analysis about this phenomenon on additively manufactured (FFF) ASA.

In this sense, this work analyses the fracture behaviour of (FFF) ASA single-edge-notch bending (SENB) fracture specimens containing U-notches of different notch radii, from 0 mm (crack-like defects) up to 2 mm, not only covering normal (cracked) fracture specimens, but also including notch-type defects that make it possible to evaluate the notch effect, i.e., how fracture resistance evolves when increasing the radius at the notch tip. The notch effect may be crucial from a structural integrity point of view, as there are materials for which a small radius in the defect tip leads to significant increases in the fracture resistance (e.g., [10,11,38,39,40,41]), and others for which the introduction of a significant radius in the defect tip does not produce a significant improvement (growth) in the fracture resistance (e.g., [38,42,43]). Finally, this research also covers the influence of printing orientation on the notch effect, analysing it for three different raster orientations: 0/90, 45/−45 and 30/−60.

The theoretical framework used to analyse the experimental results is the Theory of Critical Distances (TCD), a well-known body of knowledge that allows fracture and fatigue processes in notched materials to be analysed. Although it was first proposed around the middle of the past century [44,45], it has been widely developed over the last two decades (e.g., [38,39,40,41,42,43,46]).

Section 2 includes the description of the materials and the methods used in the analysis, Section 3 presents the results obtained and the corresponding discussion and analyses and Section 4 recapitulates the main conclusions.

## 2. Materials and Methods

**Figure 1:**
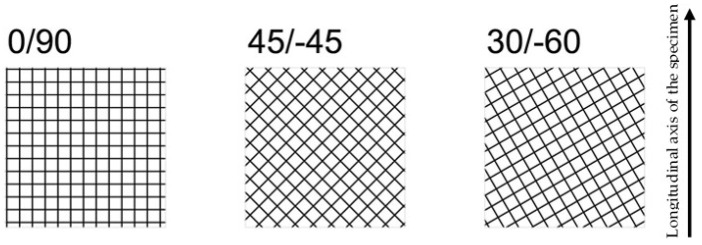
Schematic of the three different raster orientations analysed in this research. All specimens were printed in the flat position.

This research analyses the fracture behaviour and the notch effect observed in additively manufactured (FFF) ASA specimens containing U-notches. With this aim, an experimental programme composed of 72 fracture tests (SENB specimens) and 9 tensile tests was designed. The fracture specimens covered four different nominal notch radii (0 mm, 0.50 mm, 1 mm and 2 mm) and three different raster orientations (0/90, 45/−45 and 30/−60), with six specimens per combination of notch radius and raster orientation to facilitate capturing the inherent scatter of the fracture results. In the case of the tensile tests, three specimens were tested per combination, as the scatter associated with tensile results is less pronounced than in fracture tests. All specimens (fracture and tensile) were printed in the flat position, with a schematic of the raster orientations shown in Figure 1.

The ASA filaments were provided by 3DJake (Paldau, Austria). The subsequent samples were fabricated by FFF (CreatBot F430 printer, Zhengzhou, China), with the following printing parameters: layer height: 0.2 mm; line width: 0.42 mm; infill degree: 100%; printing temperature: 250 °C; bed temperature: 90 °C; printing speed: 40 mm/s. These parameters were selected as typical values found in the literature and are not intended here to be a subject of optimisation with the aim of maximising the resulting mechanical properties. The notches of the SENB specimens were machined, except for those with a notch radius of 0 mm (i.e., crack-like defects), which were generated by sawing with a razor blade. Machined defects tend to generate higher strengths than printed defects [47] and, moreover, they do not include additional anisotropy around the notch tip.

**Figure 2:**
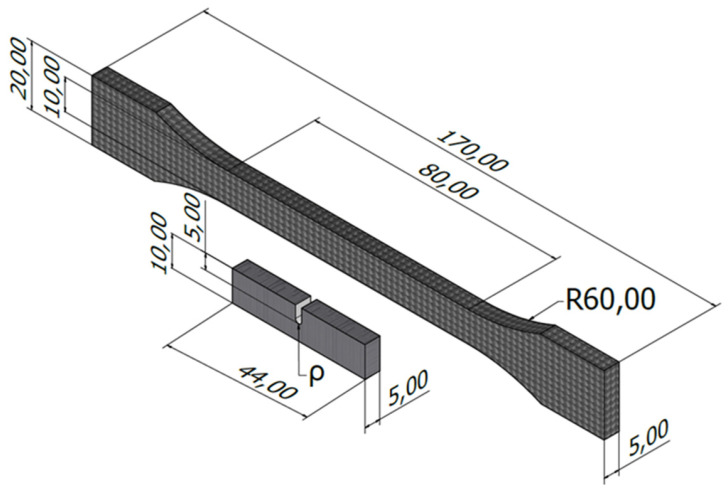
Geometry of the tensile (dog-bone) specimens and the fracture (notched SENB) specimens. Dimensions in mm.

The tensile tests were performed at room temperature following ASTM D638 [48] standard in a universal servo-hydraulic testing machine (Servosis, Madrid, Spain), with a load capacity of 5 kN, and using an axial extensometer (INSTRON, Norwood, MA, USA). The applied loading rate was 1 mm/min. Fracture tests were also performed at room temperature on SENB specimens, following ASTM D6068 [49] standard. In this case, a universal electro-mechanical machine (Zwick-Roell, Ulm, Germany) with a load capacity of 2.5 kN was employed, applying a crosshead displacement rate of 1 mm/min. A schematic of both tensile and fracture specimens is shown in Figure 2.

The fracture results will be quantified in terms of the apparent fracture toughness (K^N^_mat_), which refers to the fracture resistance developed by the material in the presence of a notch (i.e., a defect with a radius on the tip different to zero), unlike the material fracture toughness (K_mat_), which refers to the fracture resistance in the presence of crack-like defects. Subsequently, K^N^_mat_ results will be analysed by using the TCD, which comprises different methodologies, all of them characterised by the use of a material length parameter denominated the critical distance (L). In fracture analyses, L follows Equation (1) as follows:

(1) $$L=\frac{1}{\pi }{\left(\frac{K_{mat}}{\sigma_{o}}\right)}^{2}$$

where K_mat_ is the fracture toughness and σ_0_ is the material inherent strength. In those materials with linear-elastic behaviour at both the micro and the macro scales, σ_0_ coincides with the material tensile strength (σ_t_), whereas in materials with non-linear behaviour, σ_0_ requires calibration.

**Figure 3:**
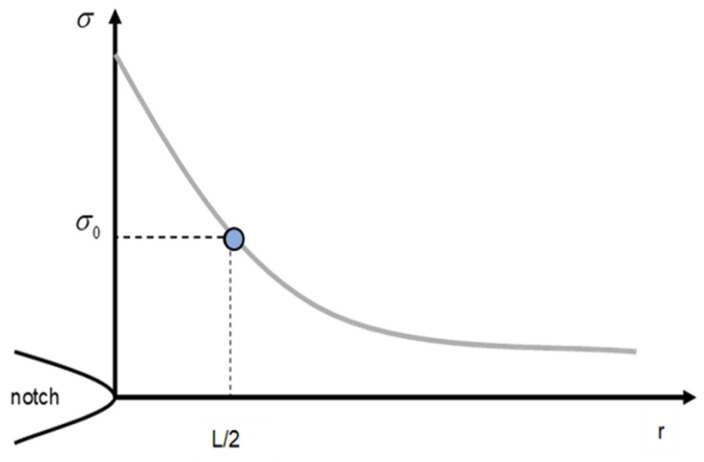
Definition of the PM methodology, based on the existing stress field at the notch tip.

The methodologies comprising the TDC are based on the stress field generated at the defect tip being analysed [38], with two of them, the Point Method (PM) and the Line Method (LM), presenting a better balance between complexity and accuracy in the predictions. The PM is the simplest approach and assumes that fracture takes place when the stress reaches inherent stress, at a distance of r_c_ from the defect tip equal to L/2 [38]. The failure criterion is as follows (see Figure 3):

(2) $$\sigma \left(\frac{L}{2}\right)=\sigma_{0}$$

The LM considers that fracture occurs when the average stress along a distance of 2L (measured from the defect tip) reaches the inherent strength (σ_0_). Thus, the fracture condition follows Equation (3) as follows:

(3) $$\frac{1}{2L}\int_{0}^{2L}\sigma \left(r\right)dr=\sigma_{0}$$

The TDC also allows notched components to be analysed in a fairly simple way, with the fracture criterion being defined by Equation (4) as follows:

(4) $$K_{I}=K_{mat}^{N}$$

In other words, if a fracture in cracked conditions occurs when the stress intensity factor (K_I_) reaches the material fracture toughness (K_mat_), the TDC provides an analogous assessment for notch-type defects, substituting the fracture toughness of the material by the corresponding apparent fracture toughness ($K_{mat}^{N}$). Moreover, $K_{mat}^{N}$ may be estimated from the combination of the Creager-Paris stress field at the notch tip [50] with the TCD fracture criteria. The resulting estimations are given by Equations (5) and (6) for the PM and LM, respectively:

(5) $$K_{mat}^{N}=K_{mat}\frac{{\left(1+\frac{\rho }{L}\right)}^{3/2}}{\left(1+\frac{2\rho }{L}\right)}$$

(6) $$K_{mat}^{N}=K_{mat}\sqrt{1+\frac{\rho }{4L}}$$

The application of these two equations is restricted to (sufficiently) slender U-notches, given that the validity range of the Creager-Paris stress distribution is restricted to this kind of stress riser. This being said, the authors have applied both equations to similar geometries to those analysed in this work, and with notch radii up to 2.0 mm (i.e., limited slenderness), obtaining reasonable results (e.g., [10,11,39]).

The research performed will be completed with a Scanning Electron Microscopy (SEM) analysis of the fracture surfaces, in order to determine the fracture micromechanisms in the different conditions (i.e., notch radius and raster orientation).

## 3. Results

### 3.1. Tensile Tests

**Figure 4:**
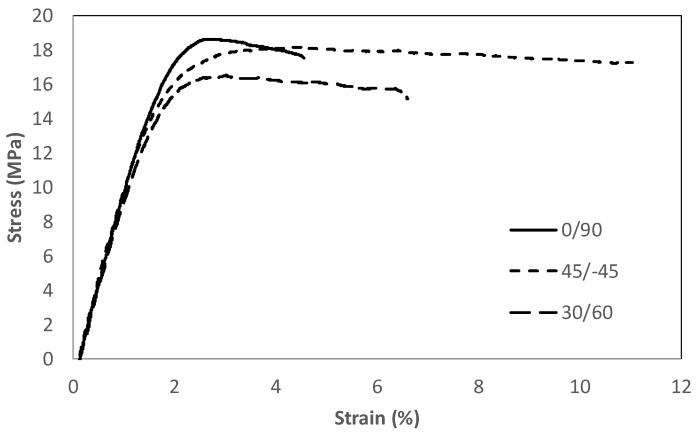
Examples of tensile curves (one per raster orientation).

The main tensile properties derived from the tensile tests are shown in Table 1, with Figure 4 showing some of the obtained tensile curves. The maximum strength is achieved in raster orientation 0/90, with the minimum being observed in orientation 30/−60. For the three raster orientations, the tensile strength (the maximum tensile stress sustained by the specimen during the test) is located at yield, which, as defined per ASTM D638 [48], corresponds to the first point on the stress–strain curve at which an increase in strain occurs without an increase in stress. Additionally, before achieving the tensile strength, the three materials lose their linear-elastic behaviour and, after yield, they develop different extents of non-linear (plastic) decreasing stress–strain behaviour up to the final break. In any case, the strength results are consistent with those reported in the literature, but clearly located in the lower bound (see Section 1 above). This, however, is not an issue here, as the objective of this research is to analyse the notch effect, and not the maximisation of tensile strength through the optimisation of printing parameters and/or the application of material post-treatments. Raster orientation 0/90, moreover, provides the lower ductility.

### 3.2. Fracture Tests

**Figure 5:**
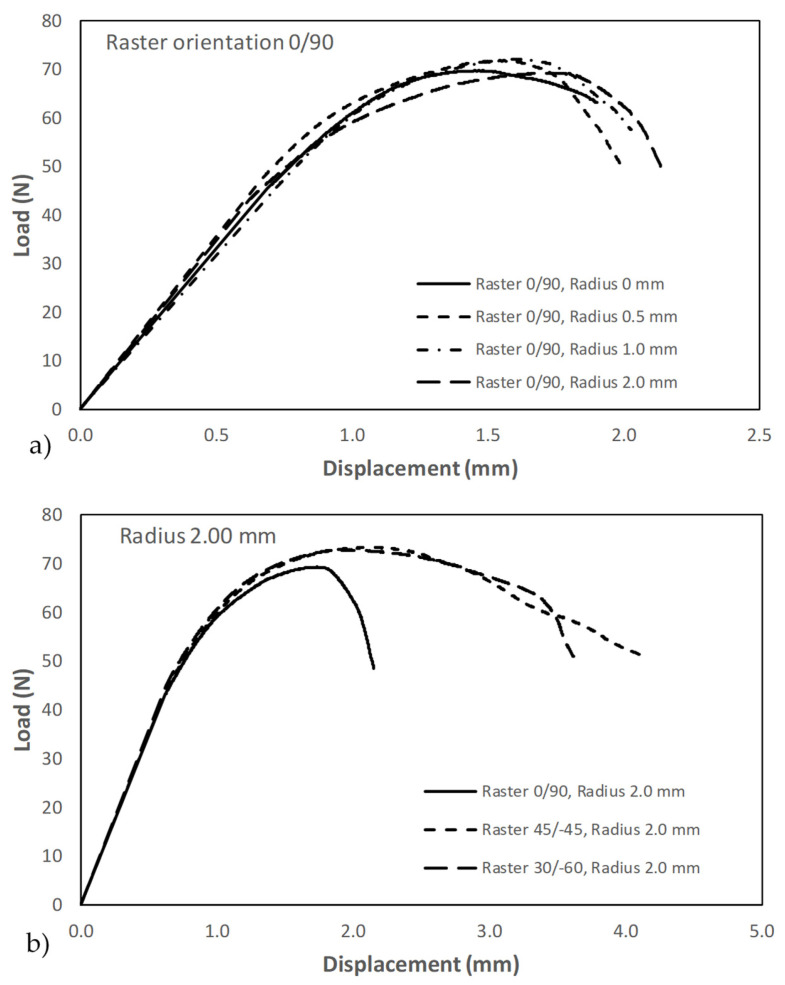
Examples of load displacement curves obtained in fracture specimens: (**a**) effect of notch radius; (**b**) effect of raster orientation.

Fracture tests were performed following ASTM D6068 standard [49], provided that the resulting load–displacement curves did not meet the linear-elastic requirements of ASTM 5045 [51]. All the load–displacement curves (see Figure 5) initially have a clearly linear-elastic behaviour, which is followed by a loss of linear-elasticity up to the corresponding maximum load of the curve. Finally, the maximum point (at which $J_{mat}^{N}$ is calculated) is followed by a decreasing load–displacement relation. Thus, and considering that no stable crack propagation was detected before the final rupture of the specimens, ASTM D6068 was used to calculate a critical value of the J integral at maximum load (P_crit_) as follows:

(7) $$J_{mat}^{N}=\frac{\eta \cdot U^{N}}{B\cdot (W-a_{0})}$$

where U^N^ is the area below the load–displacements curve up to the maximum load (see Figure 5), η is a coefficient equal to 2 in SENB specimens, B is the thickness of the specimen, W is the width of the specimen and a_0_ is the initial defect length (measured as per ASTM6068). $J_{mat}^{N}$ values were immediately converted into stress intensity factor units (K_mat_ for cracked specimens; $K_{mat}^{N}$ for notched specimens) using Equation (8) as follows:

(8) $$K_{mat}^{N}=\sqrt{\frac{J_{mat}^{N}\cdot E}{1-\upsilon^{2}}}$$

where υ is the Poisson’s ratio and E is the Young’s modulus. Figure 5 shows examples of the obtained load–displacement curves, while Table 2, Table 3 and Table 4 gather the individual results of the different tests (with the corresponding measured geometrical parameters, beyond the nominal ones). In Figure 5a, it can be observed how the notch effect is very moderate for a given raster orientation, with very similar curves for the different notch radii and with the area below the curve up to the maximum load (directly related to the fracture toughness) being slightly larger when the notch radius increases. Analogously, Figure 5b shows that, for a given notch radius, the area below the curve up to the maximum load (and then the toughness) is lower for raster orientation 0/90 and higher for raster orientation 45/−45.

**Figure 6:**
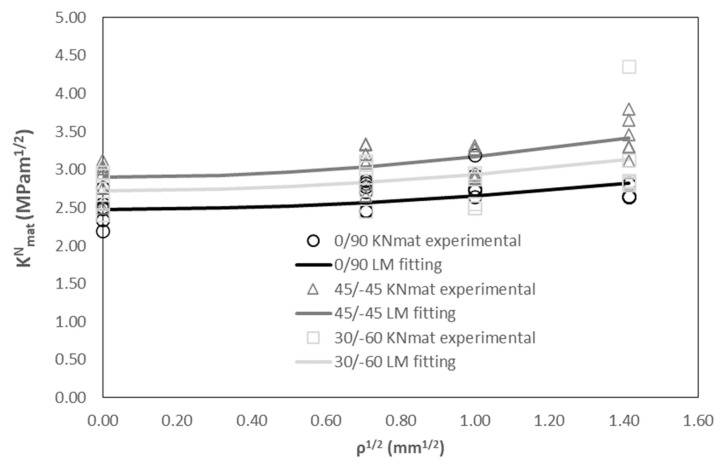
Apparent fracture toughness experimental results and LM fitting.

Figure 6 represents the evolution of the apparent fracture toughness experimental results (gathered in Table 2) with the notch radius (i.e., notch effect), together with the LM fitting (Equation (6)), with L being the fitting parameter and following the least squares approach. In addition, K_mat_ is fixed as the average value obtained in cracked conditions. In other words, the points in Figure 6 represent the individual K^N^_mat_ experimental results for the three raster orientations, whereas the lines are the corresponding best-fitting curve of Equation (6) for each orientation. The results show that raster orientation 45/−45 provides the highest values of apparent fracture toughness for the range of notch radii analysed here, including the fracture toughness itself (obtained in cracked specimens). Raster orientation 0/90 provides the lowest values, with 30/−60 providing intermediate results. This is in agreement with previous results obtained by the authors in 3D printed ABS [11] and graphene reinforced PLA (PLA-Gr) [10], but is opposite to those observed in 3D-printed PLA [10]. The results found in the literature (e.g., [51,52,53,54]) also show this trend, with raster orientation 45/−45 providing higher fracture resistance values, even for 3D-printed PLA [54], so the particular results obtained in [10] are an anomaly which would require further analysis for a better understanding. Additionally, the values of apparent fracture toughness observed in ASA (including the fracture toughness obtained in cracked conditions) are significantly lower than those observed in the other materials.

Finally, and interestingly, the fitting curves are quite parallel, implying a very similar notch effect in the three orientations. This is also observed through the obtained values of the critical distance (L), which are 1.68 mm, 1.29 mm and 1.50 mm for raster orientations 0/90, 45/−45 and 30/−60, respectively. These values are actually very similar in terms of their effect on Equation (6), considering that the term affected by L is squared, but are higher (much higher in certain cases) than those observed in the above-mentioned 3D-printed materials, with ABS [11] presenting L values of 0.92 mm (0/90 raster orientation), 0.55 mm (45/−45) and 0.46 mm (30/−60), PLA [10] having values of 0.52 mm (0/90), 0.15 mm (45/−45) and 0.67 mm (30/−60), and PLA-Gr [10] presenting values of 0.67 mm, 1.06 mm and 1.15 mm for raster orientations 0/90, 45/−45 and 30/−60, respectively. In practical terms, and provided that the critical distance (L) evaluates the material sensitivity to notch effect, these higher values of L imply that the 3D-printed ASA materials, regardless of the raster orientation, develop a lower notch effect than ABS, PLA and PLA-Gr. These observations are even more pronounced when comparing the results with those obtained in polymers obtained through conventional manufacturing processes (e.g., injection moulding), where L typically takes values in the order of 0.1 mm (e.g., PC presents values between 0.046 mm and 0.077 mm [38], PC/ABS has an L value of 0.176 mm [38], PMMA presents L values between 0.060 and 0.154 mm [38,39], etc.). Another way to understand the implication of the high values of L (i.e., low notch effect) obtained here for the 3D-printed ASA is that, when including defects or structural details in components made of this material, the fracture behaviour is quite similar to that developed when the material contains a crack-like defect, given that the apparent fracture toughness (observed in notched conditions) is not much higher than the fracture toughness (observed in cracked conditions).

### 3.3. SEM Analysis

The fractographies of both tensile and fracture specimens were analysed by using Scanning Electron Microscopy (SEM), with the aim of providing further justification of the observed mechanical behaviours.

**Figure 7:**
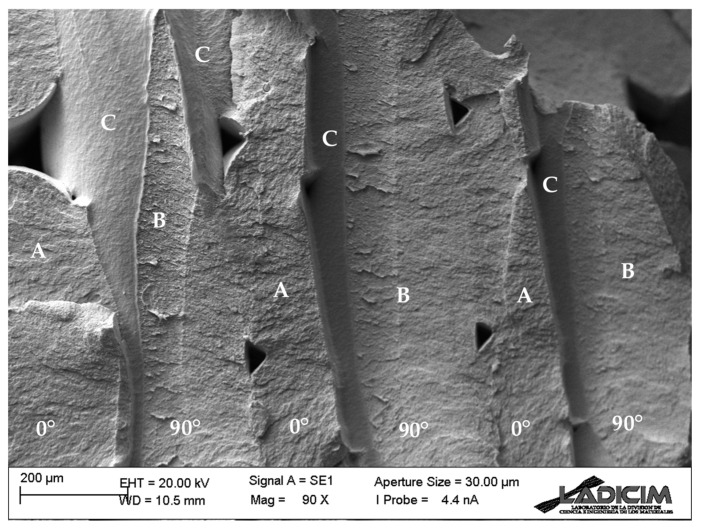
Fractography of tensile specimen with raster orientation 0/90. Areas denoted with “A” refer to 0° filaments failing through their cross section; “B” areas refer to decohesion mechanisms between 90° filaments; “C” refers to areas corresponding to the external surface of the original 90° filaments, with no contact or interaction with the adjacent filaments.

Concerning the tensile properties and the lower bound values obtained for the elastic modulus and the tensile strength (see Section 3.1), Figure 7 shows the fractography obtained in one of the tensile specimens of raster orientation 0/90. Beyond the typical pores obtained in this type of materials, it can be observed that the failure section has two clearly distinct failure conditions: filaments in the 0° orientation (aligned with the applied load) failing through their cross section (see areas denoted as “A”) and, thus, fully contributing to the overall strength, and filaments in the 90° orientation (perpendicular to the loading direction) having a much weaker behaviour. In this latter orientation, the filaments present a weak layer-to-layer adhesion with certain areas of no adhesion at all (denoted with “C”), meaning that the printing parameters are not optimised in order to obtain an optimum behaviour. These observations were general for the three raster orientations and explain the overall (low strength) macroscopic behaviour.

**Figure 8:**
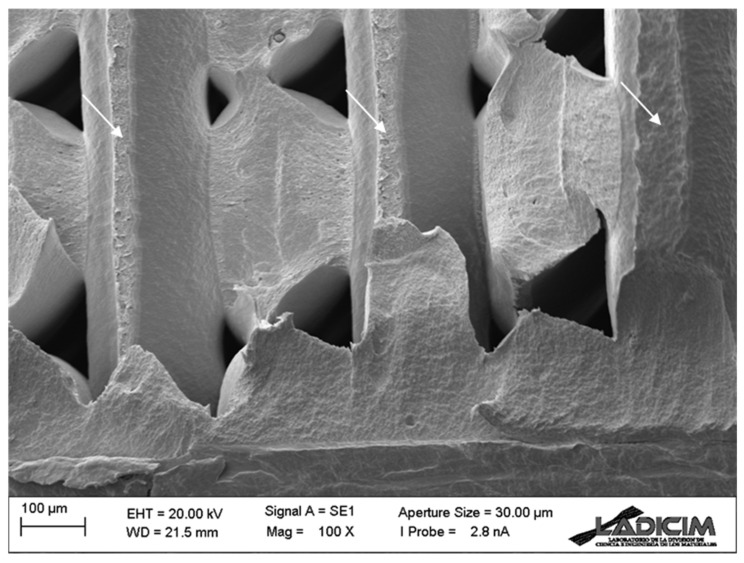
Fractography of a cracked (ρ = 0 mm) fracture specimen (test 6) with raster orientation 0/90.

**Figure 9:**
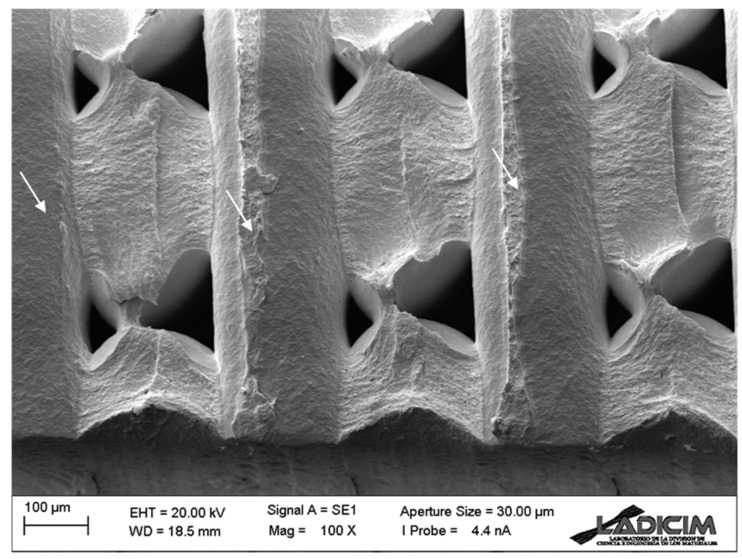
Fractography of a notched (ρ = 2.0 mm) fracture specimen (test 4) with raster orientation 0/90.

Concerning the fractographies obtained in the fracture specimens, Figure 8 and Figure 9 show, for raster orientation 0/90, an example for the two extreme notch radii considered in this work. The images show the fracture surface at the defect front (located in both cases in the lower part of the image), where crack propagation starts, showing identical micromechanisms. This justifies the little notch effect (and, consequently, the high L value) observed for these materials, given that the presence of notches is not associated with any evolution of the fracture micromechanisms towards more ductile processes, as has been reported in other materials (e.g., [39,40,42]) with much higher notch effects. In such materials, with higher notch effect (and lower L values), the fracture micromechanisms evolved when increasing the notch radius, developing increasingly ductile processes that caused a noticeable growth in fracture resistance (i.e., high notch effect). In other words, the basic contributor to the notch effect for the ASA materials analysed here is the stress relaxation caused by the notch radius. It is also observed that filaments in the 0° orientation fail through their cross section, whereas filaments in the 90° orientation contribute to fracture resistance through the decohesion (along the defect propagation plane) between adjacent filaments (see arrows in the figures).

**Figure 10:**
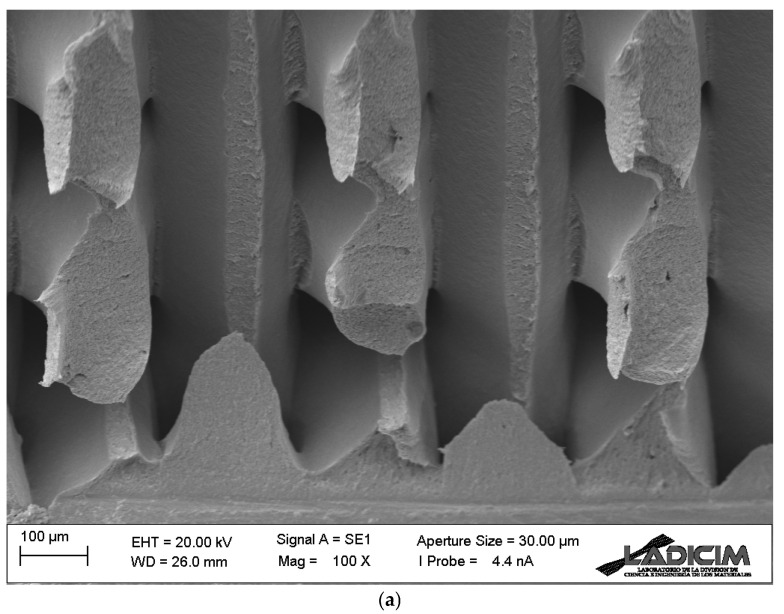

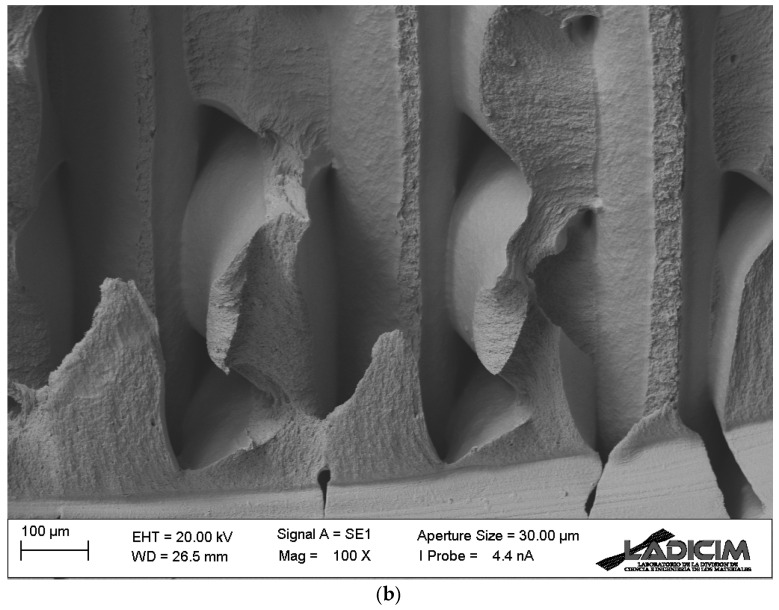
Fractography of: (**a**) cracked (ρ = 0 mm) fracture specimen (test 2) with raster orientation 45/−45; (**b**) cracked (ρ = 0 mm) fracture specimen (test 1) with raster orientation 30/−60.

**Figure 11:**
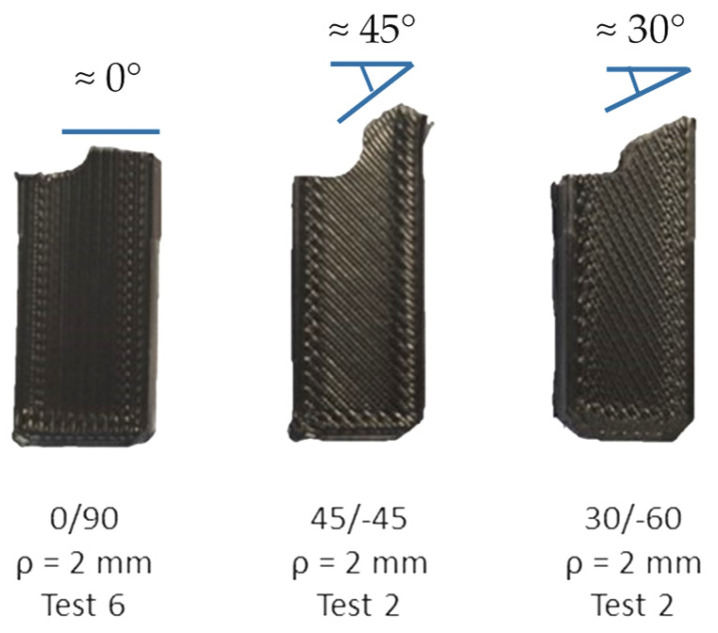
Orientation of the defect propagation planes for the different raster orientations.

The aspect of the fractographies in raster orientations 45/−45 and 30/−60 is very similar (see examples in Figure 10, with the crack fronts, again, located in the lower part of the image), although the defect propagation planes are not perpendicular to the longitudinal direction of the specimens (i.e., perpendicular to the principal bending stresses), but they basically propagate along one of the printing orientations (45° or −45° for raster orientation 45/−45; 30° or −60° for raster orientation 30/−60) and are perpendicular to the other one (−45° or 45° for raster orientation 45/−45; −60° or 30° for raster orientation 30/−60), as shown in Figure 11. These larger and less efficiently oriented propagation planes may contribute to the higher fracture resistance observed in these two raster orientations (see Figure 6).

**Figure 12:**
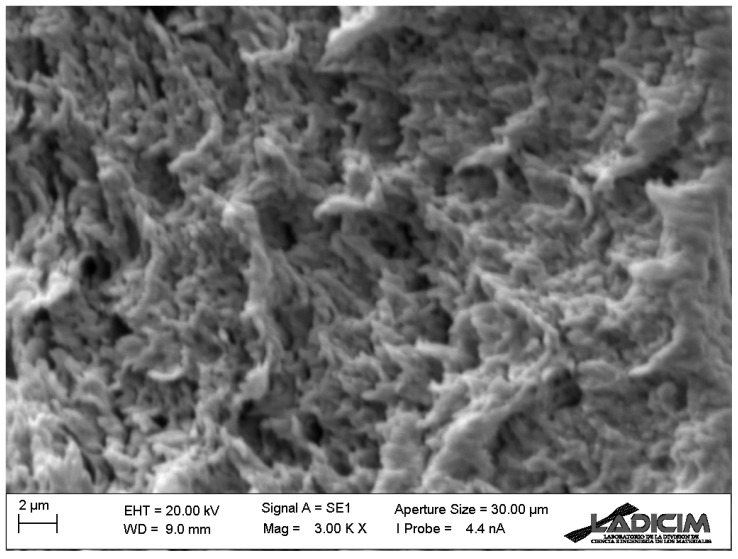
Detail of the fracture micromechanisms observed in the fracture specimens. Raster orientation 0/90, cracked specimen (ρ = 0 mm), test 6.

Finally, Figure 12 shows a detail of the fracture micromechanisms observed in all the specimens, regardless of the raster orientation and notch radius. In spite of the generally brittle appearance observed in the fracture surfaces (e.g., Figure 8 and Figure 9), non-linear (ductile) micromechanisms are observed at higher magnification, justifying the overall non-linear behaviour observed in the load–displacement curves (see Figure 5).

## 4. Conclusions

This paper analyses the notch effect in 3D-printed ASA material with three different raster orientations (0/90, 45/−45 and 30/−60) and containing notches with four different notch radii (0 mm, 0.50 mm, 1.0 mm and 2.0 mm). The characterisation specimens were printed using conventional printing parameters and were subsequently tested under tensile and fracture (SENB specimens) conditions, obtaining very moderate mechanical properties in all cases.

Concerning the notch effect, it was small if compared with other polymers and 3D printed polymers, and very similar in the three raster orientations. This implies that the material (whichever the raster orientation is) not only has moderate mechanical properties, but also that, when introducing a finite radius on the defect tip, the increase in the fracture resistance is smaller than that observed in other polymers. When quantifying the notch effect through the material critical distance (L), this parameter was 1.68 mm for raster orientation 0/90 and 1.29 mm for raster orientation 45/−45 (with raster orientation 30/−60 in between), confirming the low sensitivity of these materials to the notch effect. In addition, raster orientation 45/−45 provided the highest values of fracture resistance for the different notch radii considered in this work, with raster orientation 0/90 providing the lowest values.

SEM analyses allowed the observed mechanical behaviour to be justified. More precisely, SEM observations explained the reason for the modest mechanical properties measured in the three raster orientations, for the low (and very similar) notch effect observed in the three cases, for the non-linearity of the load–displacement curves obtained in the fracture tests, and (together with macroscopic observations for the defect propagation plane) for the higher fracture resistance of raster orientations 45/−45 and 30/−60.

Based on the findings of this work, future research will be undertaken with the aim of maximising the mechanical behaviour of 3D printed ASA, including the notch effect, by optimising the printing parameters and/or by incorporating reinforcements in the polymeric matrix (e.g., carbon fibre).

## Figures and Tables

**Table 1:** Tensile properties for ASA in each raster orientation (average and standard deviation). E: Young’s modulus; σ_t_: tensile strength; ɛ_u_: Strain under maximum load.

Raster Orientation	E (MPa)	σ_t_ (MPa)	ɛ_u_ (%)
0/90	1050 ± 66	19.4 ± 1.0	2.8 ± 0.2
45/−45	1053 ± 22	18.5 ± 0.7	4.5 ± 0.2
30/−60	990 ± 28	16.5 ± 0.1	2.8 ± 0.1

**Table 2:** Individual test results in ASA SENB notched specimens. Raster orientation 0/90.

Raster Orientation	Test	Notch Radius, ρ	W	B	a_0_	P_crit_	$K_{mat}^{N}$	$K_{mat}^{N}$ avg. ± sd.
		(mm)	(mm)	(mm)	(mm)	(N)	(MPa·m^1/2^)	(MPa·m^1/2^)
0/90	1	0	9.86	5.18	4.75	101.6	2.76	2.47 ± 0.19
0/90	2	0	9.72	5.16	4.80	67.4	2.56	
0/90	3	0	9.55	5.00	4.78	78.0	2.51	
0/90	4	0	9.64	5.01	4.90	52.5	2.20	
0/90	5	0	9.75	5.13	4.80	66.2	2.49	
0/90	6	0	9.74	4.99	4.18	69.8	2.34	
0/90	1	0.51	9.67	5.09	5.17	71.9	2.71	2.68 ± 0.18
0/90	2	0.52	9.57	5.07	5.16	70.7	2.77	
0/90	3	0.50	9.40	5.05	5.15	72.8	2.82	
0/90	4	0.49	9.73	5.10	4.99	91.7	2.86	
0/90	5	0.50	9.64	5.00	4.90	68.7	2.46	
0/90	6	0.59	9.51	5.25	4.14	67.4	2.47	
0/90	1	1.03	9.74	4.99	5.13	68.5	2.72	2.85 ± 0.22
0/90	2	1.00	9.71	5.01	5.06	72.1	2.64	
0/90	3	1.01	9.83	4.95	5.06	74.2	2.76	
0/90	4	1.02	9.75	5.04	5.19	93.4	3.19	
0/90	5	1.01	9.70	5.02	5.11	73.4	2.93	
0/90	6	1.01	9.60	5.02	5.18	-		
0/90	1	2.10	9.70	5.03	4.37	69.3	2.81	2.70 ± 0.09
0/90	2	2.11	9.63	5.05	4.37	69.0	2.65	
0/90	3	2.12	9.67	5.10	4.36	69.9	2.82	
0/90	4	2.10	9.72	5.06	4.30	69.3	2.64	
0/90	5	2.13	9.60	5.01	4.34	75.1	2.64	
0/90	6	2.10	9.69	5.12	4.35	67.9	2.65	

**Table 3:** Individual test results in ASA SENB notched specimens. Raster orientation 45/−45.

Raster Orientation	Test	Notch Radius, ρ	W	B	a_0_	P_crit_	$K_{mat}^{N}$	$K_{mat}^{N}$ avg. ± sd.
		(mm)	(mm)	(mm)	(mm)	(N)	(MPa·m^1/2^)	(MPa·m^1/2^)
45/−45	1	0	9.74	5.00	4.94	68.5	2.49	2.90 ± 0.23
45/−45	2	0	9.80	5.06	4.40	88.9	2.95	
45/−45	3	0	9.84	5.14	5.07	87.8	3.12	
45/−45	4	0	9.52	5.05	4.97	82.5	3.04	
45/−45	5	0	9.58	5.10	5.02	82.0	2.75	
45/−45	6	0	9.68	5.16	5.25	89.1	3.01	
45/−45	1	0.60	9.14	5.39	4.77	54.3	2.65	3.07 ± 0.28
45/−45	2	0.50	9.59	5.12	4.90	86.1	3.34	
45/−45	3	0.70	9.67	5.01	4.77	76.4	3.33	
45/−45	4	0.67	9.68	5.05	5.06	71.8	3.19	
45/−45	5	0.60	9.36	5.20	5.10	70.5	3.09	
45/−45	6	0.56	9.70	5.16	5.19	56.7	2.80	
45/−45	1	1.13	9.57	5.04	4.58	78.0	2.94	3.10 ± 0.20
45/−45	2	1.02	9.45	5.24	4.80	72.0	3.26	
45/−45	3	1.10	9.52	5.23	4.71	82.9	2.88	
45/−45	4	1.05	9.26	5.10	4.99	59.8	3.26	
45/−45	5	1.10	9.64	5.14	5.08	83.6	3.31	
45/−45	6	1.12	9.55	5.06	4.54	80.6	2.93	
45/−45	1	2.14	9.71	5.02	5.28	81.5	3.29	3.44 ± 0.25
45/−45	2	2.05	9.56	5.10	5.20	75.3	3.65	
45/−45	3	2.02	9.54	5.12	5.40	73.3	3.11	
45/−45	4	2.40	9.51	5.13	5.50	63.3	3.79	
45/−45	5	2.20	9.50	5.12	4.52	77.0	3.30	
45/−45	6	2.07	9.57	5.06	5.21	73.3	3.46	

**Table 4:** Individual test results in ASA SENB notched specimens. Raster orientation 30/−60.

Raster Orientation	Test	Notch Radius, ρ	W	B	a_0_	P_crit_	$K_{mat}^{N}$	$K_{mat}^{N}$ avg. ± sd.
		(mm)	(mm)	(mm)	(mm)	(N)	(MPa·m^1/2^)	(MPa·m^1/2^)
30/−60	1	0	9.76	5.14	4.66	79.8	2.40	2.72 ± 0.22
30/−60	2	0	9.67	5.18	4.99	97.5	2.97	
30/−60	3	0	9.73	5.13	5.02	66.6	2.67	
30/−60	4	0	9.64	5.21	5.16	85.5	2.78	
30/−60	5	0	9.67	5.11	4.86	71.9	2.55	
30/−60	6	0	9.85	5.14	5.17	87.8	2.92	
30/−60	1	0.40	9.64	5.13	4.58	86.0	2.45	2.88 ± 0.25
30/−60	2	0.21	9.51	5.11	4.85	79.2	2.89	
30/−60	3	0.22	9.47	5.15	4.85	68.2	3.02	
30/−60	4	0.31	9.59	5.24	5.14	78.7	2.73	
30/−60	5	0.40	9.70	5.14	5.24	86.0	3.11	
30/−60	6	0.50	9.59	5.18	5.25	73.5	3.08	
30/−60	1	1.13	9.66	5.23	4.55	67.9	2.49	2.72 ± 0.22
30/−60	2	1.10	9.41	5.22	4.76	71.7	2.87	
30/−60	3	1.00	9.45	5.23	4.69	75.7	2.55	
30/−60	4	1.15	9.38	5.17	4.98	75.2	2.95	
30/−60	5	1.12	9.60	5.23	5.38	81.2	2.91	
30/−60	6	1.05	9.57	5.22	4.59	76.5	2.49	
30/−60	1	2.12	9.66	5.06	4.29	66.2	2.84	3.13 ± 0.61
30/−60	2	2.13	9.63	5.13	4.68	84.8	3.12	
30/−60	3	2.11	9.60	5.08	4.43	74.2	2.80	
30/−60	4	2.10	9.75	5.24	4.21	88.0	4.35	
30/−60	5	2.05	9.67	5.25	4.37	72.8	2.82	
30/−60	6	2.12	9.56	5.07	4.70	66.6	2.85	

## Data Availability

The raw data supporting the conclusions of this article will be made available by the authors on request.
